# Synchrotron FTIR micro-spectroscopy for structural analysis of Lewy bodies in the brain of Parkinson’s disease patients

**DOI:** 10.1038/srep17625

**Published:** 2015-12-01

**Authors:** Katsuya Araki, Naoto Yagi, Yuka Ikemoto, Hisashi Yagi, Chi-Jing Choong, Hideki Hayakawa, Goichi Beck, Hisae Sumi, Harutoshi Fujimura, Taro Moriwaki, Yoshitaka Nagai, Yuji Goto, Hideki Mochizuki

**Affiliations:** 1 Department of Neurology, Osaka University Graduate School of Medicine, 2-2 Yamadaoka, Suita, Osaka 565-0871, Japan; 2 Japan Synchrotron Radiation Research Institute (JASRI/SPring-8), 1-1-1 Kouto, Sayo, Sayo, Hyogo 679-5198, Japan; 3 Institute for Protein Research, Osaka University, 3-2 Yamadaoka, Suita, Osaka 565-0871, Japan; 4 Center for Research on Green Sustainable Chemistry, Tottori University, 4-101 Koyamacho-minami, Tottori, Tottori 680-8550, Japan; 5 Department of Neurology, Toneyama National Hospital, 5-1-1 Toneyama, Toyonaka, Osaka 560-8522, Japan; 6 Department of Degenerative Neurological Diseases, National Institute of Neuroscience, National Center of Neurology and Psychiatry, 4-1-1 Ogawa-Higashi, Kodaira, Tokyo 187-8502, Japan

## Abstract

Lewy bodies (LBs), which mainly consist of α-synuclein (α-syn), are neuropathological hallmarks of patients with Parkinson’s disease (PD). The fine structure of LBs is unknown, and LBs cannot be made artificially. Nevertheless, many studies have described fibrillisation using recombinant α-syn purified from *E. coli*. An extremely fundamental problem is whether the structure of LBs is the same as that of recombinant amyloid fibrils. Thus, we used synchrotron Fourier transform infrared micro-spectroscopy (FTIRM) to analyse the fine structure of LBs in the brain of PD patients. Our results showed a shift in the infrared spectrum that indicates abundance of a β-sheet-rich structure in LBs. Also, 2D infrared mapping of LBs revealed that the content of the β-sheet structure is higher in the halo than in the core, and the core contains a large amount of proteins and lipids.

Lewy bodies (LBs), which mainly consist of α-synuclein (α-syn), are neuropathological hallmarks of patients with Parkinson’s disease (PD) and dementia with Lewy bodies^1^,^2^. α-syn is highly expressed in the central nervous system^3^,^4^,^5^ and erythrocytes^6^, yet its function remains obscure. Although α-syn exists *in vitro* as an intrinsically disordered monomeric protein^7^, it transforms into fibrils after incubation for 1 week to 3 months^8^,^9^. Recently, fibrils were formed in a few days by agitation at 120 to 1000 rpm at 37 °C^10^,^11^. Because these fibrils have a β-sheet-rich structure and cross-β conformation as seen with Fourier transform infrared spectroscopy (FTIR) and X-ray diffraction^12^, these fibrils are often regarded as ‘amyloid fibrils,’ which are defined biophysically as fibrillar polypeptide aggregates with cross-β conformation^13^,^14^. Although elucidating the fibrillization process of α-syn is difficult, several studies have indicated that misfolded monomers undergo self-assembly into metastable oligomeric intermediates and finally, into amyloid-like fibrils^15^,^16^. Recent epoch-making studies showed that the fibril seeds formed *in vitro* as described above can be propagated in the mouse brain^17^,^18^. The hypothesis stating that α-syn fibrils are involved in the pathogenesis of PD is under investigation *in vivo* as well as *in vitro*.

On the other hand, the secondary structure of proteins in LBs in the human brain has not been clarified. Several EM studies demonstrated that LBs contain a filamentous amyloid-like structure, which is morphologically similar to wild-type α-syn fibrils *in vitro*^19^,^20^,^21^,^22^,^23^,^24^. In addition, they showed that typical LBs had granular components in the core and radially arranged fibrils in the halo^19^,^20^,^21^,^22^,^23^,^24^. These results indicate that part of LBs is ‘amyloid fibrils’ that are pathologically characterised as depositions of protein fibrils with a specific appearance in EM. However, EM cannot provide information on the secondary structure of a protein, and thus, whether LBs have β-sheet conformation is unclear. To confirm that LBs contain amyloid fibrils, it is necessary to verify the abundance of β-sheet.

In most clinical studies, the brains of PD patients are studied with optical or fluorescence microscopy following staining. Previous staining studies revealed that pale bodies and Lewy neurites (LNs) are precursors of LBs^25^. Triple fluorolabelling for α-syn, thiazin red, and ubiquitin or neurofilament, which enables observation of the three-dimensional distribution, is useful for distinguishing LBs, pale bodies, and LNs^25^,^26^. According to these studies, the typical LBs measured in the present study are likely to be mature LBs, in which the core and halo can be clearly identified by general staining. Haematoxylin-eosin (HE) staining showed that the core is eosinophilically stained well, but the halo appears unstained^26^,^27^. In contrast, immunostaining with anti-α-syn antibody stains the halo but not the core^26^,^27^. Analysis using staining is sufficient for classical pathological differential diagnosis, but it does not provide fine structural information. Although Congo red, Thioflavin-T, and Thiazin red, which can stain Senile plaques (SPs), can be used for amyloid detection, their specificity depends on the solution and staining conditions^28^. Furthermore, LBs are immunostained by many antibodies that are raised against a native monomer^2^,^29^. In view of the fine structure, α-syn in LBs may not have a conformation abundant in β-sheet similar to amyloid fibrils of α-syn that form *in vitro*. Because LBs cannot be induced *in vivo* or *in vitro*, a technique that can detect β-sheets in real LBs from the brains of PD patients is needed to elucidate the nature of LBs.

FTIR is an established structural analysis method and is sensitive to the secondary structure of proteins (Fig. 1). An absorption maxima for α-helix (~1655 cm^−1^), β-sheet (~1630 cm^−1^), and random coil (~1645 cm^−1^) structures are included in the frequency range of the amide I band^28^,^30^. FTIR also provides information on the amount of lipids in the beam^31^. Because FTIR shows the spectrum derived from a chemical bond, it provides detailed structural information that cannot be obtained with staining and EM. However, FTIR measurement of LBs is not easy for several reasons. The main difficulty is that LBs are too small to be irradiated with an infrared beam, and their density is too low to obtain a significant signal. To overcome this, a strong and small infrared beam is required. For this reason, we used synchrotron radiation at the SPring-8 synchrotron radiation facility (Hyogo, Japan) (Fig. 2). Here, we present, to our knowledge, the first data on the secondary structure of LBs that was obtained using synchrotron Fourier transform infrared micro-spectroscopy (FTIRM). Furthermore, β-sheet mapping was performed to elucidate the process of generation of LBs.

## Results

### FTIRM of recombinant α-syn fibrils, SPs, and LBs

Figure 3 shows the FTIR spectra of recombinant α-syn fibrils (A), of brain sections from an SP of an Alzheimer’s disease (AD) patient (Fig. 3B,C), and of an LB of an 83-year-old female PD patient (Fig. 3D–F). The spectra were fitted by a model centred with Gaussian peaks at 1628, 1680 (β-sheets), 1648, and 1661 (random coils, α-helices, and others) cm^−1 ^12,30,31.

Table 1 shows the proportion of the β-sheet structures. The fibrils of α-syn purified from *E. coli* have an absorption spectrum with a broad amide I band, which has a maximum at 1628 cm^−1^ (Fig. 3A). This peak indicates the presence of β-sheet-rich proteins. The contribution of the dye that was used to identify the LB to the spectrum seems negligible because peaks that are characteristic of the dye were not observed (Fig. S1).

The spectra of brain sections from an SP of an AD patient (Fig. 3B,C) and an LB of a PD patient (Fig. 3D–F) show greater contributions from β-sheet structures than α-helical structures. Interestingly, proportion of the β-sheet was higher in the halo (63.8%) than in the core of the LB (48.4%) (Fig. 3E,F). In particular, the peak at 1628 cm^−1^, which is characteristic of β-sheet structure, in the halo (44.9%) was as high as that in the recombinant fibrils (40.9%) (Fig. 3 and Table 1). The proportion of β-sheet structures in the core of an SP was similar to that in the core of an LB (Fig. 3C,E).

### 2D FTIRM mapping of SPs

Figure 4 shows 2D mapping of components in the FTIRM spectrum for a section of an SP that was analysed using the method of Liao *et al.*^31^ and Bousset *et al.*^12^. The protein map was obtained by the sum of the absorbance at 1540 cm^−1^ and 1640 cm^−1^, whereas that of the β-sheet was obtained by the proportion of the fitted peaks at 1628 cm^−1^ and 1680 cm^−1^ and lipids by the 2850 cm^−1^ peak. The amount of total proteins was high in the centre of the core of SPs (Fig. 4, Protein), and the proportion of β-sheet structures was also high in the centre of the core of SPs (Fig. 4, β-sheet). On the other hand, the amount of lipids was high around the core (Fig. 4, Lipid). The result of the distribution of lipids was consistent with a previous report^31^, which used a frozen section. Because the present study used a deparaffinised section, the result shows that the lipids in an SP were not lost during the deparaffinising process.

### 2D FTIRM mapping of LBs

Figure 5 shows 2D mapping of components of the FTIRM spectra for sections of the brain of a PD patient with typical mature LBs. The amount of total proteins was high in the core of LBs (Fig. 5, Protein). This result is consistent with the images obtained with HE staining and EM. In contrast, the proportion of β-sheet structures was low in the core and high in the halo (Fig. 5, β-sheet). This suggests that the halo includes more fibril-like structure than the core. On the other hand, lipids tended to be concentrated in the core of LBs (Fig. 5, Lipid). Among the twenty LBs studied in this work, eight did not show a concentrated protein-rich region. Since such a structure is inconsistent with EM images of typical LBs, we consider this due to technical difficulties associated with the sample preparation: thickness may not be uniform in the section, or part of the section may have been deformed or split during sectioning, staining or dehydration. Seven (58%) in the well-prepared twelve images showed the same distribution of protein, β-sheet and lipid as described above. The remaining five (42%) showed a low content of lipid and a rather high content of β-sheet in the core (Fig. S2).

## Discussion

Using FTIRM, we found that the secondary structure of LBs in the brain of a PD patient *in vivo* was similar to the β-sheet conformation of recombinant α-syn fibrils *in vitro*. As far as we are aware, these are the first data on the secondary structure of LBs *in vivo*. There have been similar reports on SPs of the brain of an AD patient^31^,^32^,^33^, but a study on LBs, which are smaller than SPs, requires a higher spatial resolution.

Interestingly, in typical LBs, which are relatively large, the region with a high content of β-sheet structures is the halo, whereas the amount of total protein is high in the core. Although the core is well stained in EM and likely to have high protein density, it does not necessarily indicate the presence of a high amount of fibrils and β-sheets in the core. Our FTIR data for a typical mature LB showed not only that the proportion of β-sheets was higher in the halo than in the core, but also that proteins with different secondary structures were present in both regions. Our results indicate that the filamentous structure in the halo, which is visualized by EM, includes a high amount of β-sheets which are typically found in recombinant α-syn fibrils. On the other hand, the core has a high amount of proteins but might mainly consist of denatured proteins rather than β-sheet structures.

In this study, atypical LBs were also found in the brain of all three PD patients (Fig. S2). Since the thickness of the section is similar to the size of LBs and the sections are cut in randomly, images may vary depending on which part of the LB is included in the section. In addition, variations in the time course and the formation process of LBs may exist even in each patient. We speculate that some of the atypical LBs may be immature LBs or LNs. Therefore, in the present paper, we report mainly the results for typical mature LBs, which included more than half of the reliable twelve mapping images.

The core of typical LBs was found with FTIRM to include a high concentration of proteins (Fig. 5, Protein). This result is consistent with the observation that the core is eosinophilically stained well with HE staining. On the other hand, the core of LBs contains less β-sheet structures than the halo (Fig. 5, β-sheet), although the proportion of β-sheet structures in the core was higher than that of normal brain tissues. Although α-syn is the major component of LBs, an immunohistochemical study has shown that LBs contain more than 90 proteins^27^. Because the core is not stained well with immunostaining with an anti-α-syn antibody^2^,^31^, there may be many denatured proteins other than α-syn in the core of typical LBs. The fact that LBs are immunostained with ubiquitin^34^ supports this hypothesis. On the other hand, the halo of typical LBs has a high content of β-sheet structures that are comparable to recombinant α-syn fibrils. The halo, which has been shown with EM to contain fibres^19^,^20^,^21^,^22^,^23^,^24^, is likely to consist of amyloid fibrils of α-syn. This structure resembles a capsule that entraps waste products, suggesting that LBs prevent diffusion of denatured proteins into the cytoplasm and play a protective role for nerve cells.

In typical LBs, the distribution of lipids was high in the core (Fig. 5, Lipid). This result is consistent with a report that the core of LBs is stained with the lipid-soluble fluorescent dye rhodamine B^35^. As lipid droplets have not been observed in LBs, we believe the lipids are forming membranes. Because α-syn is known to bind to lipids and this binding causes an α-helical structure^36^,^37^, α-syn in the core may be bound to lipids and have higher content of α-helical structure than that in the halo.

Because LBs mainly consist of aggregated α-syn and soluble recombinant α-syn can be readily fibrillised, we believe that they share the same structure. On the basis of this tacit understanding, many studies regarding fibrillization of recombinant α-syn have been reported^38^. However, the most important problem still remains that LBs are not formed *in vitro* or *in vivo* by recombinant α-syn. Therefore, clinical research is needed to determine whether the structure of LBs from patient samples is similar to that of recombinant α-syn fibrils. From this viewpoint, our result is simple yet crucial to support the validity of amyloid studies for PD *in vitro*. However, LBs are not simple aggregates of α-syn fibrils but consist of several key elements. Therefore, further detailed structural analysis of LBs from the brains of PD patients will be essential for elucidating the nature of LBs.

Our FTIRM measurement technique, which has already been established, is likely to be useful as a tool for neuropathology analysis. However, our study also has some limitations. FTIRM only observes the sum of all of the contributions from the various proteins. Because the peaks from the biological materials are too broad for unique determination, each peak was determined as previous reports^12^,^30^,^31^. The peaks of 1628 cm^−1^ and 1680 cm^−1^ are characteristic of β-sheets but the peaks of 1648 cm^−1^ and 1661 cm^−1^ are derived from not only α-helical structure but also random coils and others. Therefore, to correctly interpret the result of FTIRM, it is necessary to combine them with those of other measurements, such as staining, EM, X-ray diffraction and Nuclear Magnetic Resonance. Furthermore, because the present FTIRM measurements were limited to LBs from only three patients, we cannot elucidate variations in the structure of LBs. Studies on samples from more patients may allow classification of LBs into several types based on the distribution of the β-sheets. In addition, to fully understand the nature of amyloid formation, measuring aggregates other than those in SPs and LBs will be important. In the brains of patients with neurodegenerative diseases, several types of aggregates such as LNs, pale bodies, and glial cytoplasmic inclusions (GCIs), have been identified with staining. GCIs, which consist mainly of α-syn, are characteristic aggregates in the brains of multiple system atrophy patients^39^. Uchihara *et al.* demonstrated a method to distinguish LBs from GCIs with silver staining^40^. Clarifying the cause of such differences is crucial for elucidating the pathogenesis of neurodegenerative diseases. Many amyloid-related diseases other than neurodegenerative diseases exist, including transthyretin of familial amyloid polyneuropathy^41^ and islet amyloid polypeptide associated with the disease mechanism of type II diabetes^42^. An interesting report has shown that amyloid fibrils derived from semen-derived enhancer of virus infection drastically enhance HIV infection^43^. Although we cannot find the reports of structure analysis *in vivo* for these proteins, some studies reported the detailed dynamic structure not only in solution but also in association with model membranes^44^,^45^,^46^,^47^. It is interesting that the reported structures and aggregation process are similar to those of α-syn and amyloid β which are related to neurodegenerative disease.

Our FTIRM approach has a potential for elucidating the pathology of many amyloid-related diseases. However, since the laboratory FTIR instrument does not have sufficient brightness, FTIRM measurement on brain tissues requires synchrotron radiation. This has been limiting the use of this technique particularly for medical researchers. However, since many synchrotron radiation facilities have been recently constructed worldwide, more researchers have a better access to FTIRM for biological samples now.

## Methods

All experimental protocols were approved by the Ethical Review Board at Osaka University Graduate School of Medicine and were performed in accordance with the Ethical Guidelines for Clinical Research of the Ministry of Health, Labour and Welfare of Japan. Informed consent was previously obtained from all subjects.

### Preparation of recombinant α-syn and fibrils

Human wild-type α-syn cDNA was cloned into the bacterial expression vector pET23a(+). α-syn proteins were expressed in *Escherichia coli* BL21 (DE3) (Novagen, Darmstadt, Germany) and purified as described previously^48^. Purified human α-syn (5 mg/ml) was incubated at 37 °C in a shaking incubator (200 rpm) in 50 mM Tris–HCl, 150 mM NaCl, pH 7.5, for 1 week. Fibril formation was confirmed with the Thioflavin-T (Wako) binding assay^49^ using a CORONA SH-9000 Lab fluorescence microplate reader. Aliquots were mixed with 5 μM Thioflavin-T in PBS, and fluorescence intensities were monitored at 480 nm with excitation at 450 nm. In addition, fibril formation was morphologically confirmed with EM (Fig. S3).

For FTIRM, 50 μl of a solution that included fibrils was placed on calcium fluoride (CaF_2_). Before measurement, these samples were allowed to dry at room temperature.

### Preparation of brain sections for FTIRM measurement

Brain tissue samples from three patients (83-year-old female, 76-year-old male and 74-year-old female) with neuropathologically confirmed PD and from a 75-year-old male patient with AD pathology were used for our measurement. The samples were fixed in 4% buffered formaldehyde and embedded in paraffin according to routine tissue processing for pathological examination. For each sample, 10-μm-thick sections were cut and deposited on CaF_2_. Tissue sections from the patient’s brain with AD pathology were stained with Congo red. Tissue sections from the PD patient’s brain were incubated with the primary antibody, pSyn#64 (anti-human phosphorylated α-syn (Ser129) monoclonal antibody, Wako), overnight, followed by incubation with horseradish peroxidase-conjugated anti-mouse IgG (The Dako EnVision+ System, Dako). Final staining was completed with incubation in 3,3′-diaminobenzidine + substrate-chromogen for 5–10 min to allow formation of a brown-coloured precipitate at the antigen site. The stained tissue was then examined with optical microscopy to confirm the presence and locations of SPs and LBs. Before measurement, these samples were allowed to dry at room temperature.

### Synchrotron FTIRM measurement

**Figure 2:**
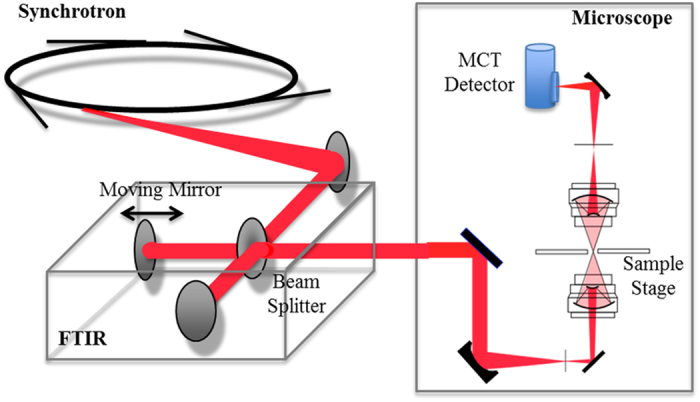
Optical layout of the microspectroscopic station at BL43IR. The infrared synchrotron light is injected into the FTIR (BRUKER VERTEX70) interferometer, and the light then goes to the microscope (BRUKER HYPERION2000). Infrared light transmitted through a sample is detected by an MCT (HgCdTe) detector.

Synchrotron FTIR spectra and images were collected at the infrared beamline BL43IR at the SPring-8 synchrotron radiation facility (Hyogo, Japan) (Fig. 2). The brilliance of infrared synchrotron radiation (IR-SR) at BL43IR is more than two orders of magnitude higher than that of the laboratory source in the fingerprint region of 2000–1000 cm^−1 ^50. A Fourier transform infrared (FTIR: BRUKER VERTEX70) spectrometer was used with IR-SR as the infrared source. A tissue sample on the adjustable motorised x–y mapping stage can be observed with an optical microscope (BRUKER HYPERION2000). A rectangular region 100 μm × 100 μm, including amyloid deposits or LBs, was mapped with an aperture size of 7 μm × 7 μm and 3–5 μm steps in the horizontal and vertical directions. Interferograms were acquired with 400 scans, and signals were averaged and Fourier transformed to generate a spectrum with a nominal resolution of 3 cm^−1^.

### FTIRM spectral analysis

**Figure 3:**
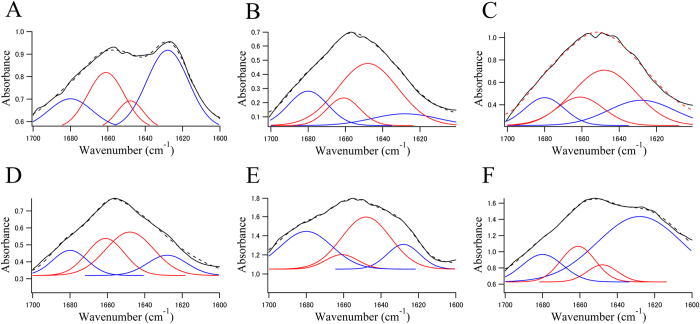
The solid black line in each panel shows an FTIRM spectrum (amide I region) obtained from (A) fibrils of α-syn expressed in *E. coli*, (B) normal brain tissue from the patient with AD, (C) the core of an SP, (D) normal brain tissue from the brain of the patient with PD, (E) the core of an LB, and (F) the halo of an LB. Blue and red lines represent contributions of β-sheet structures and non-β-sheet structures (random coils, α-helices, and others), respectively. The dotted line represents the fitted curve. Data were fitted using a Gaussian species model centred at 1628, 1680 (β-sheets, blue line), 1648, and 1661 (random coils, α-helices, and others, red line) cm^−1 ^12,30,31.

**Figure 4:**
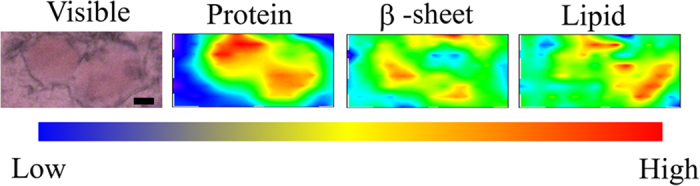
Typical visible and FTIR images of an SP in brain tissue derived from the patient who had AD pathology. Shown from left to right are a microscope image, the amount of total proteins, the proportion of β-sheet structures, and the amount of lipids. The colour bar indicates low (blue) to high (red) contents. The area in the visible image was scanned with 5-μm steps (16 × 8 pixels = 80 × 40 μm^2^). Scale bar, 10 μm. Protein-rich regions correspond well to the regions stained with Congo red. The proportion of β-sheet structures is high in the core of the plaque. Lipids exist around the core of the plaque.

**Figure 5:**
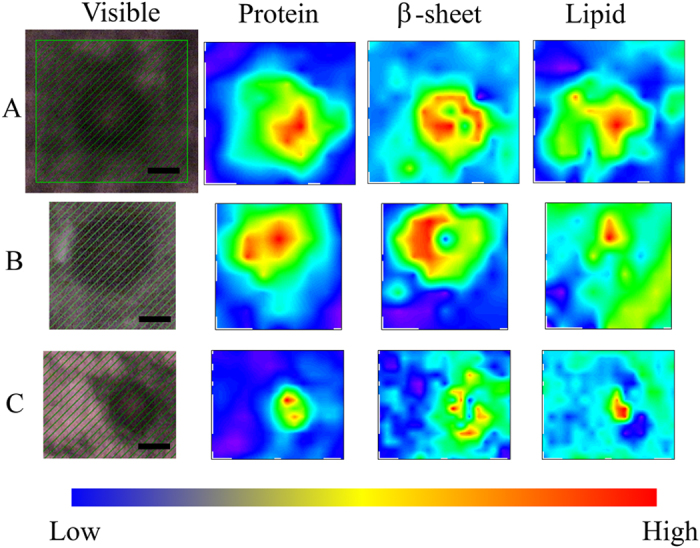
Visible and FTIR images of typical LBs in the substantia nigra of the midbrain derived from the 83-year-old female PD patient. Different LBs and different scanning steps are shown. Shown from left to right are a microscope image, the amount of total proteins, the proportion of β-sheet structures, and the amount of lipids. The colour bar indicates low (blue) to high (red) contents. The area shaded with green was scanned with 3- to 5-μm steps. (**A**) 4-μm step, 12 × 11 pixels = 48 × 44 μm^2^. (**B**) 5-μm step, 8 × 9 pixels = 40 × 45 μm^2^. (**C**) 3-μm step, 14 × 17 pixels = 42 × 51 μm^2^. Scale bar, 10 μm.

Analysis was performed using Igor Pro software (version 6.01, WaveMetrics). Total protein distribution was evaluated by calculating the sum of the absorbance at 1540 cm^−1^ and 1640 cm^−1^. The proportion of β-sheet structures was analysed from a curve fit for the FTIR spectra ranging from 1700 cm^−1^ to 1600 cm^−1^. Spectrum data were fitted using four Gaussian species centred at 1628 cm^−1^ and 1680 cm^−1^ (β-sheets), and 1648 cm^−1^ and 1661 cm^−1^ (random coils, α-helices, and others) as in previous reports^12^,^30^,^31^. The secondary structure cannot be uniquely determined from the peaks at 1648 cm^−1^ and 1661 cm^−1^. During the fitting procedure, the peak height was free, whereas the width at half-height was maintained at <25 cm^−1^. A reasonable fit was obtained as shown in Fig. 3. Lipid distribution was based on the area of the symmetric CH_2_ band at 2850 cm^−1^ (2858–2848 cm^−1^, baseline 3000–2750 cm^−1^). From the spectra acquired in the mapping experiments, the integrated area of the two Gaussian functions representing β-sheets was calculated for each spectrum, and after smoothing between adjacent pixels, the result was plotted as a function of the position to produce a contour plot for β-sheets. The integrated area of four Gaussian functions was used as total proteins for the plot of β-sheets.

## Additional Information

**How to cite this article**: Araki, K. *et al.* Synchrotron FTIR micro-spectroscopy for structural analysis of Lewy bodies in the brain of Parkinson's disease patients. *Sci. Rep.* **5**, 17625; doi: 10.1038/srep17625 (2015).

## Supplementary Material

Supplemental Figures

## Figures and Tables

**Figure 1:**
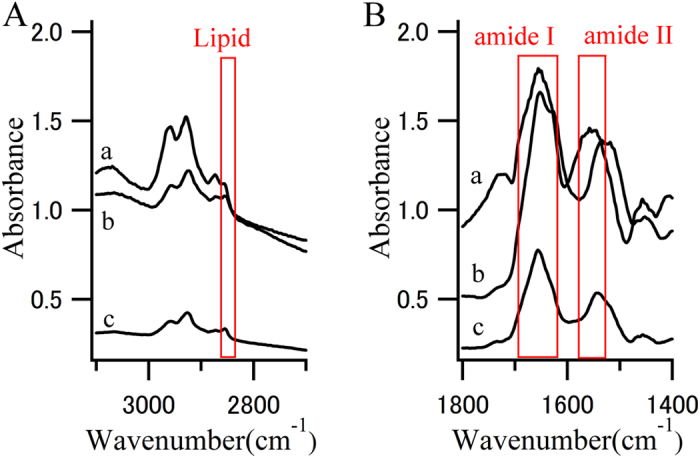
FTIR spectra. (**A**) CH stretch region and (**B**) amide I and II region from the (a) core of an LB, (b) halo of an LB, and (c) normal brain tissue. Red boxes represent characteristic absorption region for lipids or proteins.

**Table 1:** FTIR deconvolution.

Wavelength (cm^−1^)	1628	1648	1661	1680	β-sheet
A: the fibrils of recombinant α-syn (Area %)	40.9	14.4	29.2	15.5	56.4
B: Normal brain tissue of AD (Area %)	8.4	46.0	20.3	25.3	33.7
C: the core of SP (Area %)	22.1	34.5	21.8	21.6	43.7
D: Normal brain tissue of PD (Area %)	16.1	34.5	29.4	20.0	36.1
E: the core of LB (Area %)	19.2	40.4	11.2	29.2	48.4
F: the halo of LB (Area %)	44.9	11.7	24.5	18.9	63.8

A–F correspond to the panels in Fig. 3. The deconvolution indicates that the β-sheet content of the halo of an LB (63.8%) is higher than that of the core of an LB (48.4%) and SP (43.7%). In particular, the proportion of the area under the peak at 1628 cm^−1^, which represents the amount of β-sheet structures, in the halo is as high as that in the fibrils of recombinant α-syn.
