# Supplementary material for: Synchrotron FTIR micro-spectroscopy for structural analysis of Lewy bodies in the brain of Parkinson’s disease patients
Source: Sci Rep. 2015 Dec 1;5:17625. doi: 10.1038/srep17625 (PMC4664933; doi:10.1038/srep17625)

Supplementary Information for

Synchrotron FTIR micro-spectroscopy for structural analysis of Lewy bodies in the brain of Parkinson's disease patients

Katsuya Araki[1], Naoto Yagi[2*], Yuka Ikemoto[2], Hisashi Yagi[3,4], Chi-Jing Choong[1], Hideki Hayakawa[1], Goichi Beck[1], Hisae Sumi[1], Harutoshi Fujimura[5], Taro Moriwaki[2], Yoshitaka Nagai[6], Yuji Goto[3], and Hideki Mochizuki[1*]

[1]Department of Neurology, Osaka University Graduate School of Medicine, 2-2 Yamadaoka, Suita, Osaka 565-0871, Japan

[2]Japan Synchrotron Radiation Research Institute (JASRI/SPring-8), 1-1-1 Kouto, Sayo, Sayo, Hyogo 679-5198, Japan

[3]Institute for Protein Research, Osaka University, 3-2 Yamadaoka, Suita, Osaka 565-0871, Japan

[4]Center for Research on Green Sustainable Chemistry, Tottori University, 4-101 Koyamacho-minami, Tottori, Tottori 680-8550, Japan

[5]Department of Neurology, Toneyama National Hospital, 5-1-1 Toneyama, Toyonaka, Osaka 560-8522, Japan

[6]Department of Degenerative Neurological Diseases, National Institute of Neuroscience, National Center of Neurology and Psychiatry, 4-1-1 Ogawa-Higashi, Kodaira, Tokyo 187-8502, Japan

Supplementary Figure S1

FTIRM spectra for the halo of LBs (solid line) and the primary and secondary antibody with DAB (dye, dashed line): The unique peaks of dye, such as 1100 cm^{-1} and 2570 cm^{-1}, are not seen in the spectrum for the halo of LBs.

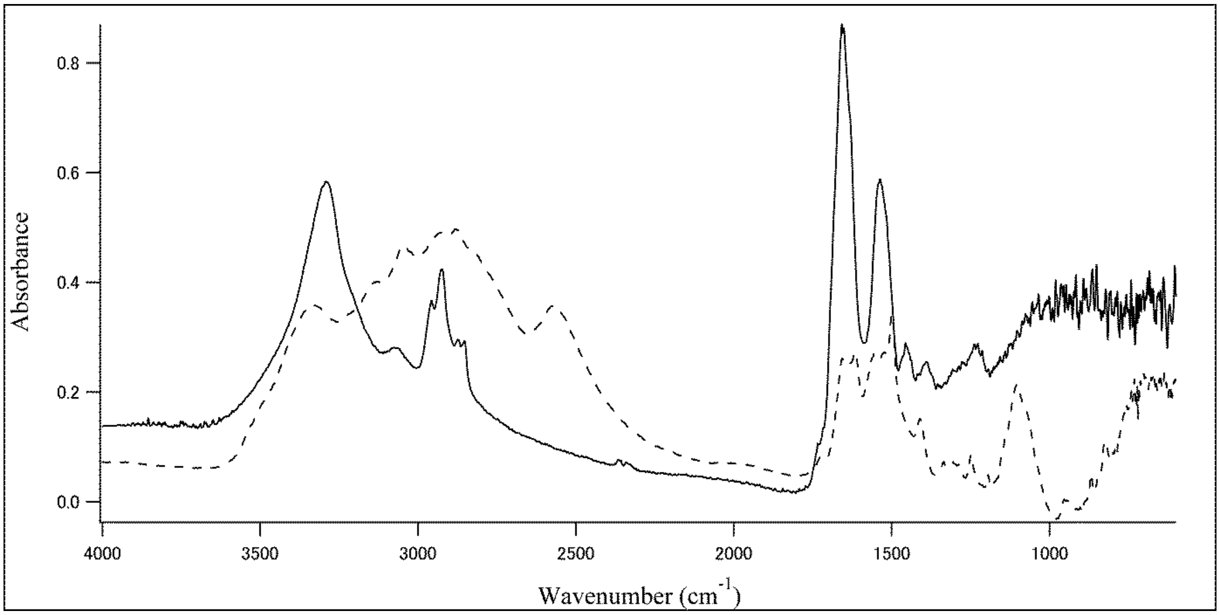

Supplementary Figure S2

Visible and FTIR images of atypical LBs in the substantia nigra of the midbrain derived from the PD patient. We have measured a total of 20 pieces of LBs with FTIRM. Five (42 %) in the well-prepared 12 mapping images showed that the content of β-sheet was not high in the halo. (A) 4-μm step, 11 × 10 pixels = 44 × 40 μm^2. (B) 4-μm step, 23 × 10 pixels = 92 × 40 μm^2. Scale bar, 10 μm.

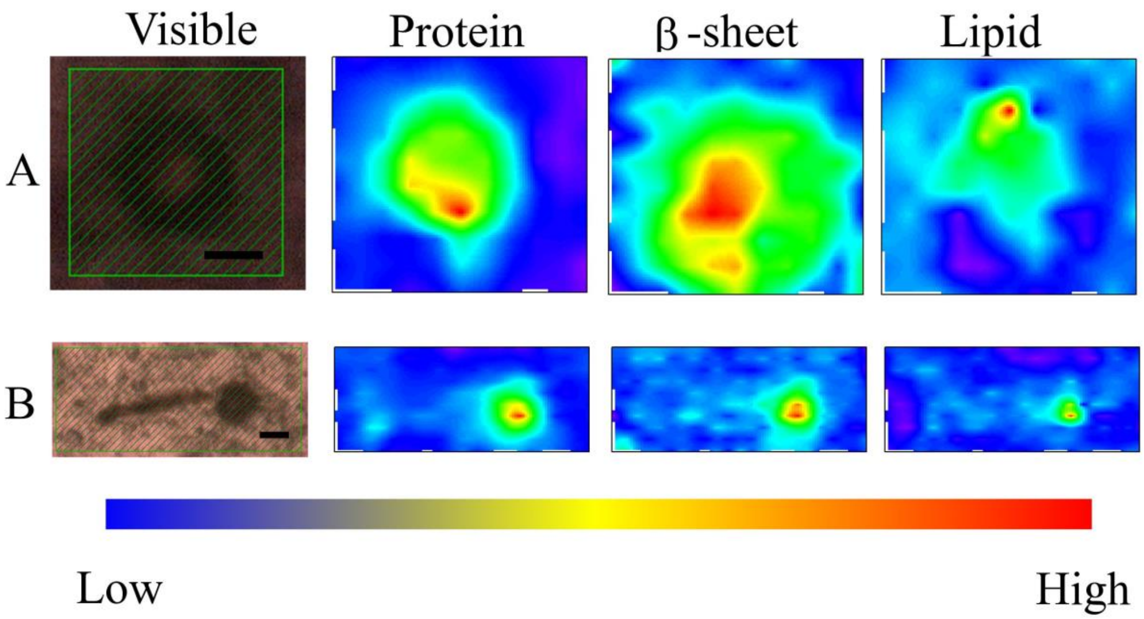

Supplementary Figure S3

Electron microscopic image of α-syn fibrils. Scale bar, 300 nm.

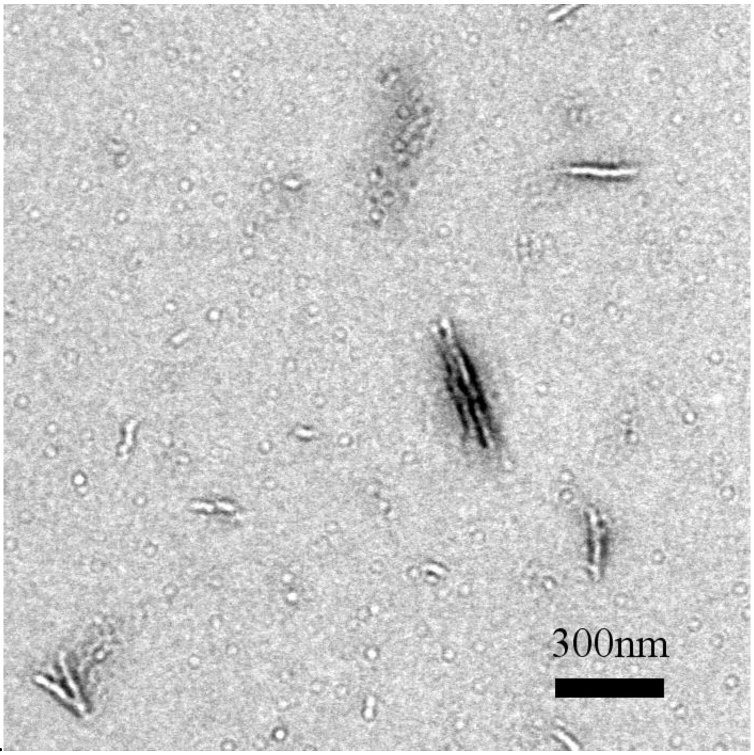

Supplement: Supplemental Figures [file srep17625-s1.pdf]
